# Diagnostic performance of radiologists in distinguishing post-COVID-19 residual abnormalities from interstitial lung abnormalities

**DOI:** 10.1007/s00330-024-11075-x

**Published:** 2024-09-23

**Authors:** Jong Eun Lee, Hyo-Jae Lee, Gyeryeong Park, Kum Ju Chae, Kwang Nam Jin, Eva Castañer, Benoit Ghaye, Jane P. Ko, Helmut Prosch, Scott Simpson, Anna Rita Larici, Jeffrey P. Kanne, Thomas Frauenfelder, Yeon Joo Jeong, Soon Ho Yoon

**Affiliations:** 1 Department of Radiology and Research Institute of Radiology, Asan Medical Center, Seoul, Korea; 2 Department of Radiology, Chonnam National University Hospital Gwangju, Gwangju, Korea; 3 Department of Radiology, Research Institute of Clinical Medicine of Jeonbuk National University-Biomedical Research Institute of Jeonbuk National University Hospital, Jeonju, Korea; 4 Department of Radiology, Seoul National University-Seoul Metropolitan Government Boramae Medical Center, Seoul, Korea; 5 Department of Radiology, Parc Taulí Hospital Universitari, Institut d’Investigació i Innovació Parc Taulí (I3PT-CERCA), Universitat Autònoma de Barcelona, Sabadell, Spain; 6 Department of Radiology, Cliniques Universitaires Saint Luc, Catholic University of Louvain, Brussels, Belgium; 7 Department of Radiology, NYU Langone Health, NYU Grossman School of Medicine, New York, NY USA; 8 Department of Biomedical Imaging and Image-Guided Therapy, Medical University of Vienna, Vienna General Hospital, Vienna, Austria; 9 Department of Radiology, Hospital of the University of Pennsylvania, Philadelphia, PA USA; 10 Department of Diagnostic Imaging and Oncological Radiotherapy, Department of Radiological and Hematological Sciences, A. Gemelli University Polyclinic Foundation IRCCS, Catholic University of the Sacred Heart, Rome, Italy; 11 Department of Radiology, University of Wisconsin School of Medicine and Public Health, Madison, WI USA; 12 Institute of Diagnostic and Interventional Radiology, University Hospital Zurich, Zurich, Switzerland; 13 Department of Radiology, Research Institute for Convergence of Biomedical Science and Technology, Pusan National University Yangsan Hospital, Pusan National University School of Medicine, Yangsan, Korea; 14 Department of Radiology, Seoul National University College of Medicine, Seoul National University Hospital, Seoul, Korea

**Keywords:** COVID-19, Lung diseases, Interstitial, Diagnostic imaging, Tomography, X-ray computed

## Abstract

**Objective:**

Distinguishing post-COVID-19 residual abnormalities from interstitial lung abnormalities (ILA) on CT can be challenging if clinical information is limited. This study aimed to evaluate the diagnostic performance of radiologists in distinguishing post-COVID-19 residual abnormalities from ILA.

**Methods:**

This multi-reader, multi-case study included 60 age- and sex-matched subjects with chest CT scans. There were 40 cases of ILA (20 fibrotic and 20 non-fibrotic) and 20 cases of post-COVID-19 residual abnormalities. Fifteen radiologists from multiple nations with varying levels of experience independently rated suspicion scores on a 5-point scale to distinguish post-COVID-19 residual abnormalities from fibrotic ILA or non-fibrotic ILA. Interobserver agreement was assessed using the weighted κ value, and the scores of individual readers were compared with the consensus of all readers. Receiver operating characteristic curve analysis was conducted to evaluate the diagnostic performance of suspicion scores for distinguishing post-COVID-19 residual abnormalities from ILA and for differentiating post-COVID-19 residual abnormalities from both fibrotic and non-fibrotic ILA.

**Results:**

Radiologists’ diagnostic performance for distinguishing post-COVID-19 residual abnormalities from ILA was good (area under the receiver operating characteristic curve (AUC) range, 0.67–0.92; median AUC, 0.85) with moderate agreement (κ = 0.56). The diagnostic performance for distinguishing post-COVID-19 residual abnormalities from non-fibrotic ILA was lower than that from fibrotic ILA (median AUC = 0.89 vs. AUC = 0.80, *p* = 0.003).

**Conclusion:**

Radiologists demonstrated good diagnostic performance and moderate agreement in distinguishing post-COVID-19 residual abnormalities from ILA, but careful attention is needed to avoid misdiagnosing them as non-fibrotic ILA.

**Key Points:**

***Question*** *How good are radiologists at differentiating interstitial lung abnormalities (ILA) from changes related to COVID-19 infection?*

***Findings*** *Radiologists had a median AUC of 0.85 in distinguishing post-COVID-19 abnormalities from ILA with moderate agreement (κ* *=* *0.56).*

***Clinical relevance*** *Radiologists showed good diagnostic performance and moderate agreement in distinguishing post-COVID-19 residual abnormalities from ILA; nonetheless, caution is needed in distinguishing residual abnormalities from non-fibrotic ILA.*

## Introduction

By March 2024, 747.7 million people, equivalent to approximately 10% of the global population, were confirmed to be infected with SARS-CoV-2 [1]. Moderate to severe COVID-19 sometimes results in persistent CT findings in the lungs, known as post-COVID-19 residual abnormalities [2, 3]. As the global cumulative burden of SARS-CoV-2 infection continues to rise, it will become increasingly common to incidentally encounter these residual abnormalities in daily practice. Although the long-term prognostic implications of these abnormalities are unknown, the 15-year trajectory of the original SARS-CoV suggests that they are likely to remain stable [4]. Furthermore, pulmonary fibrosis resulting from acute respiratory distress syndrome, one of the causes of post-COVID-19 residual abnormalities, is known to be more stable compared to other progressive interstitial lung diseases (ILDs) [5].

Meanwhile, interstitial lung abnormalities (ILA) have recently garnered attention among researchers. ILA can manifest in fibrotic or non-fibrotic forms and potentially progress to pulmonary fibrosis. ILA frequently involves subpleural areas, especially in the lower lobes [6], which are also often affected by COVID-19 [7]. Although distinguishing between ILA and post-COVID-19 residual abnormalities can be challenging because their findings may resemble each other, differentiating between these two conditions remains important, considering their potentially varying long-term outcomes [8]. If a history of moderate to severe COVID-19 is not proactively collected during CT interpretation (a practice that is clinically impractical), radiologists may need to distinguish these entities solely based on CT findings. To date, radiologists’ ability to distinguish these conditions is underexplored. Therefore, this study aimed to evaluate the diagnostic performance of radiologists in distinguishing post-COVID-19 abnormalities and ILA.

## Materials and methods

This retrospective study was approved by the institutional review board of the participating institution (CNUH-2023-259), which waived the requirement for informed consent.

### Study design and case collection

**Fig. 1:**
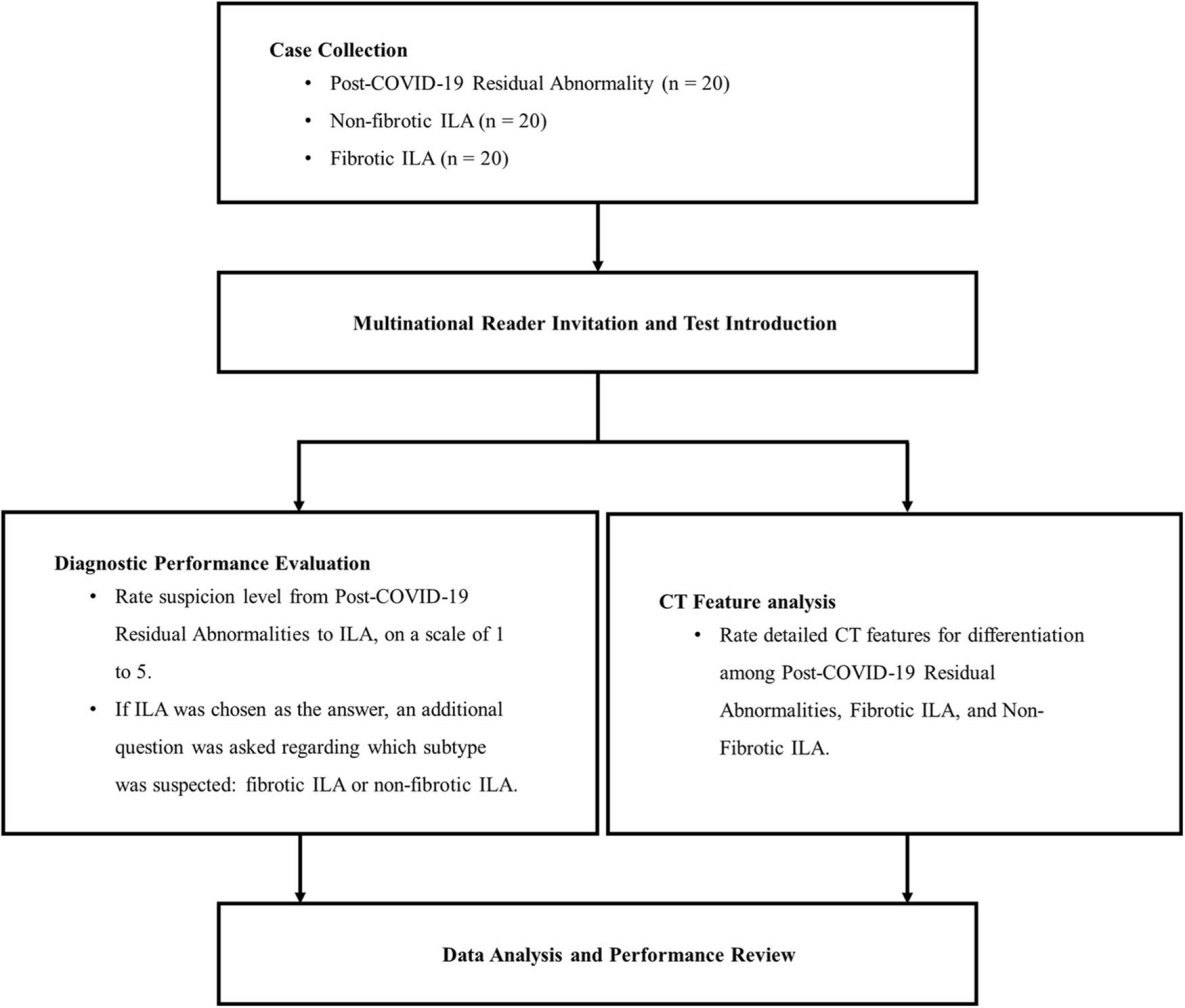
Flow diagram of the study

For this multi-reader, multi-case study, we retrospectively searched an institutional database registered in the Korean Imaging Cohort of COVID-19, spanning from January 2019 to December 2023. This study included 60 age- and sex-matched subjects with chest CT scans, consisting of 40 cases of ILA (20 fibrotic and 20 non-fibrotic) and 20 cases of post-COVID-19 residual abnormalities. The cases of ILA were collected during the period from January 2019 to December 2020, thereby excluding any history of SARS-CoV-2 infection, and were selected according to the definitions outlined in the Position Paper from the Fleischner Society [9]. Meanwhile, the cases of post-COVID-19 residual abnormalities were collected during the period from January 2022 to December 2023, with a proven history of prior SARS-CoV-2 infection and imaging evidence of previous COVID-19 pneumonia confirmed at least 6 months prior. To assemble the case collection, two thoracic radiologists, J.E.L. and S.H.Y., with 12 and 20 years of experience, respectively, selected 60 representative cases for each category (20 cases of post-COVID-19 residual abnormalities, 20 cases of fibrotic ILA, and 20 cases of non-fibrotic ILA) from the database through consensus. The methodology and case selection process are illustrated in a flow diagram (Fig. 1).

Clinical information, including age, sex, smoking history, and time since the last infection (in cases of post-COVID-19 residual abnormalities), was also collected from the medical records (Table 1).

**Table 1:** Baseline characteristics of the patient study group

Variables	Post-COVID-19 residual abnormalities (*n* = 20)	Fibrotic ILA (*n* = 20)	Non-fibrotic ILA (*n* = 20)	*p-*value
Age (y)	66.7 ± 11.2	66 ± 7.5	65.4 ± 8.1	0.90
Sex				
Male	11 (55.0)	15 (75.0)	14 (70.0)	0.37
Female	9 (45.0)	5 (25.0)	6 (30.0)	
Smoking history				
Current	1 (5.0)	7 (35.0)	7 (35.0)	0.13
Former smoker	5 (25.0)	5 (25.0)	4 (20.0)	
Never smoker	14 (70.0)	8 (40.0)	9 (45.0)	
Time since prior infection (mos)	12.8 ± 7.0	NA	NA	NA

Continuous variables are reported as mean ± standard deviation and were compared between groups using analysis of variance. Categorical variables are presented as the number of patients with percentages in parentheses and were compared between groups using chi-square test

### Image evaluation

#### Diagnostic performance evaluation

**Fig. 2:**
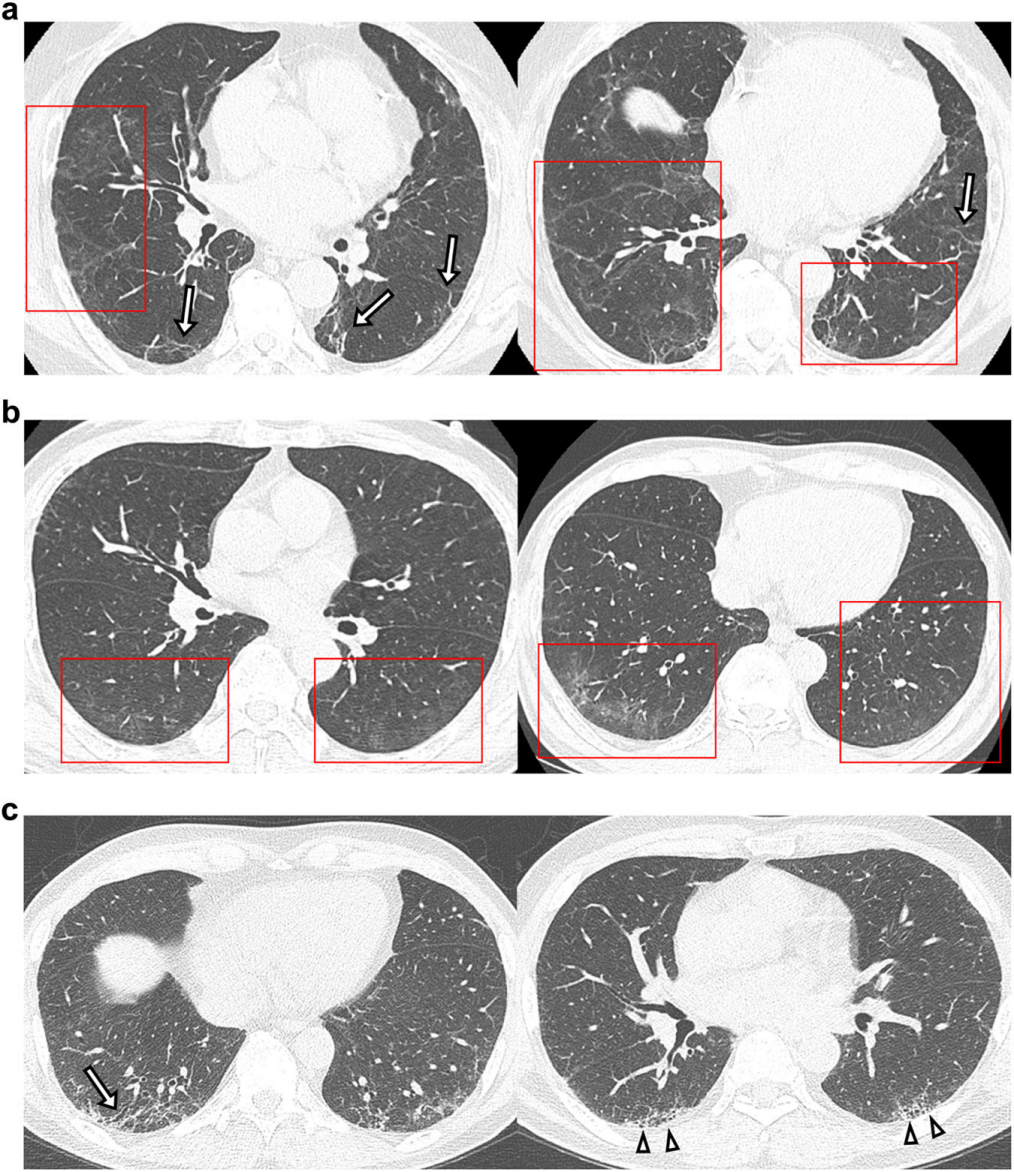
**a** Representative case of post-COVID-19 residual abnormalities. Unenhanced HRCT images show extensive GGO as the predominant finding, with a peribronchovascular distribution. Perilobular opacities (red squares) and parenchymal bands (arrows) are also present. **b** Representative case of non-fibrotic ILA. HRCT images show GGO as the predominant finding, with a subpleural and basal distribution (red squares). **c** Representative case of fibrotic ILA. HRCT images show reticulation as the predominant finding, with a subpleural and basal distribution. Traction bronchiolectasis (arrow) and non-emphysematous cysts (arrowheads) are also present

Fifteen multinational radiologists, including seven more-experienced readers (Y.J.J., E.C., B.G., J.P.K., H.P., S.S., A.R.L., 21–33 years of experience) and eight less-experienced readers (J.H.L., G.P., J.P.K., K.N.J., K.J.C., T.F., S.H.Y., J.E.L., 8–20 years of experience), were recruited for the study. Most of the radiologists were COVID-19 experts involved in the ongoing development of a multi-society post-COVID-19 management guideline, representing the Society of Thoracic Radiology, the European Society of Thoracic Imaging, and the Asian Society of Thoracic Radiology. A comprehensive training session was conducted to ensure a standardized approach to the evaluation process. Each radiologist was provided with PowerPoint (Microsoft) slides containing two representative images per lung zone, totaling six images, of post-COVID-19 residual abnormalities, fibrotic ILA, and non-fibrotic ILA. Apart from the imaging slides, clinical information, such as a history of previous SARS-CoV-2 infection, was not provided. The slides served to remind them of the imaging features and to explain the review methods using a 5-point scale for answers (Fig. 2 and Supplementary Fig. S1). Radiologists then independently assigned suspicion levels to the cases on a 5-point scale, distinguishing post-COVID-19 residual abnormalities from ILA. When ILA was identified, the readers were further prompted to determine the ILA subtype, choosing between fibrotic and non-fibrotic categories. The 5-point scale used to distinguish post-COVID-19 residual abnormalities and ILA was defined as follows: a score of 5 indicated a definite presence of post-COVID-19 residual abnormalities; a score of 4 suggested a probable presence; a score of 3 was deemed indeterminate; a score of 2 suggested probable ILA; and a score of 1 indicated definite ILA. For improved diagnostic precision, a binary scale was adopted, in which scores of 5 and 4 were classified as indicative of post-COVID-19 residual abnormalities, whereas scores of 1 to 3 were categorized as ILA. To assess the diagnostic performance in distinguishing post-COVID-19 residual abnormalities from ILA, evaluations were conducted on 20 cases of post-COVID-19 residual abnormalities and 40 cases of ILA. Furthermore, to evaluate the diagnostic performance based on the subtype of ILA, 20 cases of post-COVID-19 residual abnormalities, 20 cases of fibrotic ILA, and 20 cases of non-fibrotic ILA were each assessed.

#### CT feature analysis

Separate from the diagnostic performance evaluation, we analyzed CT features to gain insights into the features that radiologists used to distinguish between these two conditions. Three dedicated radiologists (J.E.L., K.J.C., K.N.J., with 12, 14, and 16 years of chest CT scan interpretation experience, respectively) conducted a consensus interpretation of the CT images, evaluating the CT features of post-COVID-19 residual abnormalities, fibrotic ILA, and non-fibrotic ILA in detail. For the consensus reading, the majority response was adopted as the result. The evaluated CT features encompassed the total extent of lesions in the whole lung (qualitatively estimated as either exceeding 25% or being 25% or less), distribution patterns (subpleural, peribronchovascular, basal predominance, and posterior predominance (either present or absent)), dominant findings (either ground-glass opacities (GGO) or reticulation predominance), and other findings, including the presence or absence of non-emphysematous cysts, bronchial dilation, perilobular opacities, parenchymal bands, and mosaic attenuation.

### Statistical analyses

Interobserver agreement for each reader was assessed against the consensus results (most frequent answers) of all readers using the κ statistics for both the 5-point and binary scales. For the 5-point scale, weighted κ was adopted due to its ordinal nature. The levels of agreement were classified as: κ ≤ 0.20, indicating poor agreement; κ = 0.21–0.40, signifying fair agreement; κ = 0.41–0.60, denoting moderate agreement; κ = 0.61–0.80, reflecting substantial agreement; and κ > 0.80, representing almost perfect agreement. The diagnostic performance in differentiating post-COVID-19 abnormalities was evaluated using receiver operating characteristic (ROC) curve analysis, and the area under the ROC curve (AUC) was calculated. The AUC values were compared between subgroup analyses of post-COVID-19 residual abnormalities versus fibrotic ILA and post-COVID-19 residual abnormalities versus non-fibrotic ILA using the two-independent-sample *t*-test. In addition, the sensitivity, specificity, and accuracy of the binary scale were computed. Categorical detailed CT features were compared among the three groups using the chi-square test. A *p*-value of less than 0.05 was considered to indicate a statistically significant difference. All statistical analyses were performed by J.E.L., who has previous experience performing statistical analyses in our previous works using SPSS version 28.0 (IBM).

## Results

### Patient characteristics

The mean age of the 60 subjects was 66.5 ± 7.5 years, with a male predominance (40 male and 20 female). The percentage of current smokers was higher in the fibrotic and non-fibrotic ILA groups than in the post-COVID-19 residual abnormalities group; however, this difference did not reach statistical significance (*p* = 0.13). For subjects with post-COVID-19 residual abnormalities, the mean duration since the last infection was 12.8 ± 7.0 months. The brief information on patient demographics is summarized in Table 1.

### Interobserver agreement

Interobserver agreement among all readers (*n* = 15) demonstrated moderate concordance on the 5-point scale (κ = 0.56; 95% CI: 0.50, 0.62) and the binary scale (κ = 0.64; 95% CI: 0.57, 0.71). Stratification by experience revealed that the more-experienced readers (*n* = 7) exhibited better agreement on the 5-point scale (κ = 0.59) than the less-experienced readers (*n* = 8) (κ = 0.54), but slightly lower agreement on the binary scale (κ = 0.63 vs. κ = 0.65). However, there were no significant differences in interobserver agreement between the more- and less-experienced readers (*p* = 0.40 and 0.78, respectively). The details of interobserver agreement for each reader are summarized in Table 2 and Supplementary Table S1.

**Table 2:** Interobserver agreement among readers

Variables	Interobserver agreement	
	5-point scale	Binary scale
**Average Kappa**		
All readers (*n* = 15)	0.56 (0.50, 0.62)	0.64 (0.57, 0.71)
^a^More-experienced group (*n* = 7)	0.59 (0.48, 0.70)	0.63 (0.50, 0.76)
^b^Less-experienced group (*n* = 8)	0.54 (0.46, 0.62)	0.65 (0.55, 0.76)

Data in parentheses are 95% confidence interval

^a^ More-experienced readers had 21–33 years of chest CT interpretation experience

^b^ Less-experienced readers had 8–20 years of chest CT interpretation experience

### Diagnostic performance

**Fig. 3:**
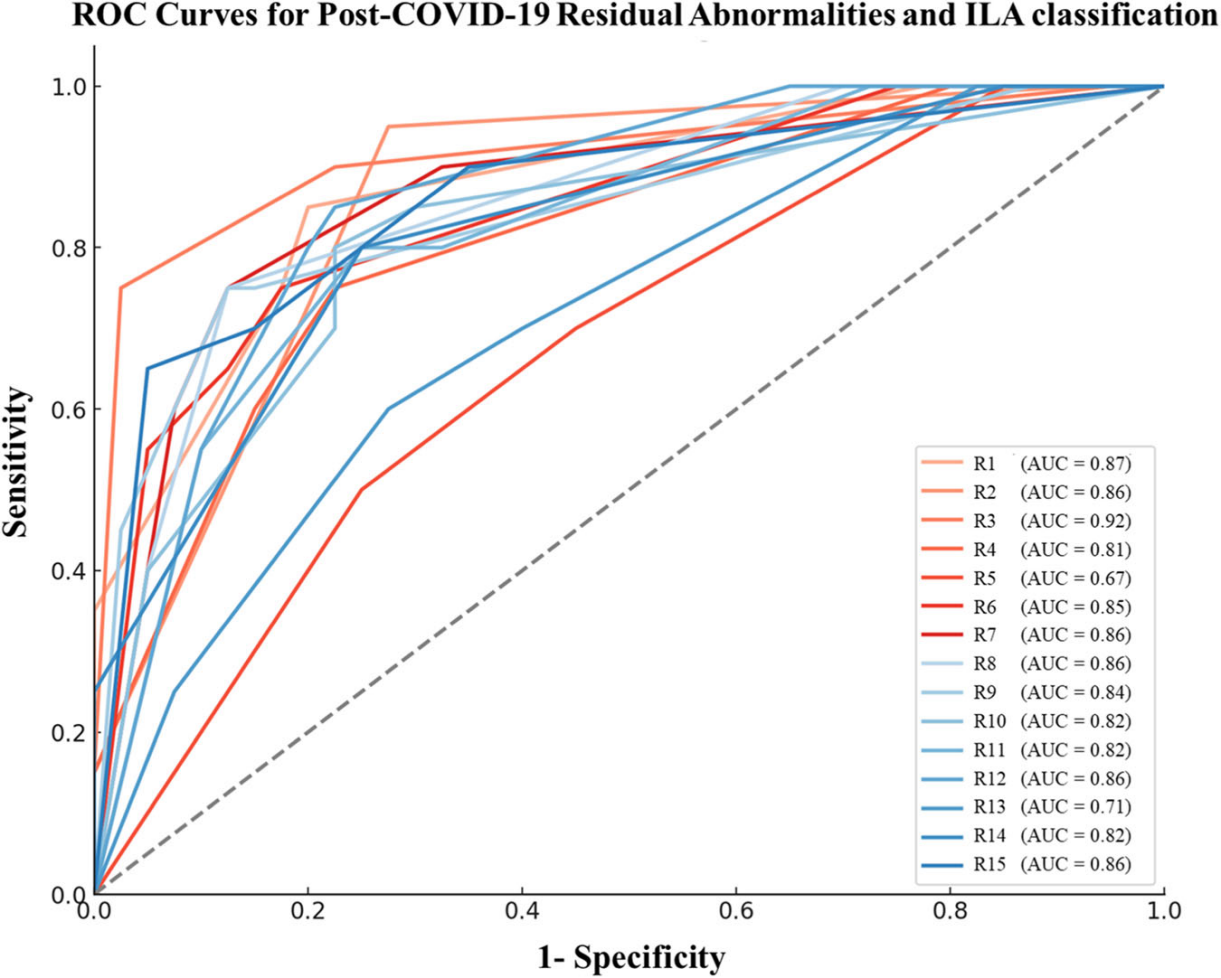
Receiver operating characteristic (ROC) curves for the classification of post-COVID-19 residual abnormalities and interstitial lung abnormalities (ILA) for each reader. Each curve represents the performance of a different reader, and the legend indicates the area under the curve (AUC) value for each reader’s ROC curve. Higher AUC values indicate better performance in distinguishing between the two conditions. The diagonal gray line represents the chance level, which is the expected performance of a random classifier

When using the 5-point scale for distinguishing post-COVID-19 residual abnormalities from ILA, the AUC values for all 15 readers ranged from 0.67 to 0.92, with a median of 0.85. The subgroup analysis revealed that the median AUC for distinguishing post-COVID-19 residual abnormalities from fibrotic ILA was higher than that for distinguishing post-COVID-19 residual abnormalities from non-fibrotic ILA (median AUC, 0.89 vs. 0.80; range, 0.68–0.94 vs. 0.56–0.89; *p* = 0.003) (Table 3). Similarly, the average AUC for differentiating post-COVID-19 residual abnormalities from fibrotic ILA was greater than that for distinguishing post-COVID-19 residual abnormalities from non-fibrotic ILA (average AUC, 0.87 vs. 0.79; *p* = 0.003). The more-experienced readers’ average AUC was significantly higher when comparing post-COVID-19 residual abnormalities with fibrotic ILA than when comparing post-COVID-19 residual abnormalities with non-fibrotic ILA (average AUC, 0.89 vs. 0.78; *p* = 0.03). However, this significant difference was not observed in less-experienced readers (*p* = 0.07). The details of the diagnostic performance for each reader are summarized in Fig. 3, Supplementary Tables S2–S4.

**Table 3:** Comparison of diagnostic performance of differentiating post-COVID residual abnormalities from ILA

Variables	Post-COVID-19 residual abnormalities vs. ILA	Post-COVID-19 residual abnormalities vs. Fibrotic ILA	Post-COVID-19 residual abnormalities vs. Non-fibrotic ILA	*p-*value^a^
**Median AUC**	0.85 (0.67, 0.92)	0.89 (0.68, 0.94)	0.80 (0.56, 0.89)	0.003
**Average AUC**				
All readers (*n* = 15)	0.83 (0.80, 0.86)	0.87 (0.84, 0.91)	0.79 (0.74, 0.84)	0.003
^b^More-experienced group (*n* = 7)	0.83 (0.76, 0.91)	0.89 (0.84, 0.94)	0.78 (0.68, 0.88)	0.03
^c^Less-experienced group (*n* = 8)	0.82 (0.78, 0.87)	0.86 (0.79, 0.92)	0.79 (0.75, 0.83)	0.07

Data are medians, and data in parentheses are ranges

^a^ The *p*-value is used to compare the average AUC for distinguishing post-COVID-19 residual abnormalities from fibrotic ILA, and for distinguishing post-COVID-19 residual abnormalities from non-fibrotic ILA

^b^ More-experienced readers had 21–33 years of chest CT interpretation experience

^c^ Less-experienced readers had 8–20 years of chest CT interpretation experience

**Fig. 4:**
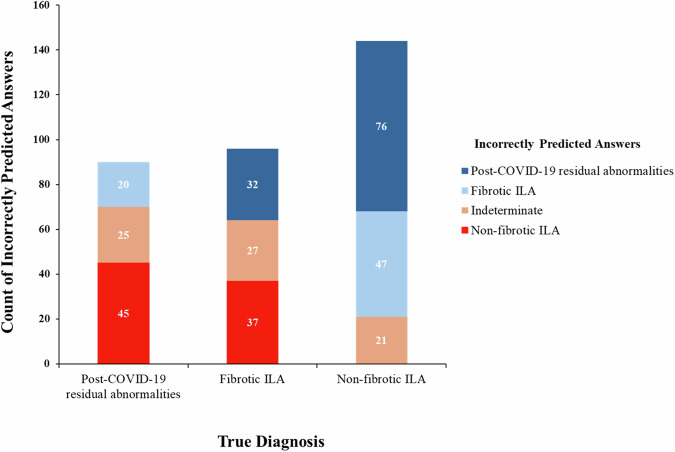
Bar graph showing the distribution of incorrect answers provided by each reader. When the true diagnosis was post-COVID-19 residual abnormalities, the most common incorrect answer given by the readers was “non-fibrotic ILA.” When the true diagnosis was non-fibrotic ILA, the most common incorrect answer was “post-COVID-19 residual abnormalities”

When the true diagnosis was post-COVID-19 residual abnormalities, the most common incorrect answer given by the readers was “non-fibrotic ILA”, accounting for 50% of incorrect answers, followed by indeterminate ILA at 28% and fibrotic ILA at 22%. Conversely, when the true diagnosis was non-fibrotic ILA, the most common incorrect answer was “post-COVID-19 residual abnormalities”, comprising 53% of incorrect answers, followed by fibrotic ILA at 32% and indeterminate ILA at 15% (Fig. 4).

### Detailed CT features

Several distinct differences in CT features were observed between post-COVID-19 residual abnormalities and both types of ILA (Table 4). Lesions involving more than 25% of the lung were more frequent in post-COVID-19 residual abnormalities than in fibrotic and non-fibrotic ILA (30% vs. 0% and 5%, respectively; *p* = 0.03). Subpleural distribution was less common in post-COVID-19 cases than in fibrotic and non-fibrotic ILA (45% vs. 100% and 95%, respectively; *p* < 0.001), while peribronchovascular involvement occurred more frequently in post-COVID-19 cases (75% vs. 5% and 25%, respectively; *p* < 0.001). Basal and posterior predominance patterns were found less frequently in post-COVID-19 abnormalities than in either type of ILA (basal: 35% vs. 90% and 85%, *p* < 0.001; posterior: 50% vs. 95% and 85%, respectively; *p* = 0.002). GGO predominance was more frequently observed in post-COVID-19 and non-fibrotic ILA than in fibrotic ILA (60% and 80%, respectively, vs. 15%, *p* < 0.001). Perilobular opacities, parenchymal bands, and mosaic attenuation were more common in post-COVID-19 abnormalities than in either type of ILA (perilobular opacities: 75% vs. 5% and 20%, respectively; *p* < 0.001; parenchymal bands: 85% vs. 15% and 20%, respectively; *p* < 0.001; mosaic attenuation: 15% vs. 0% for both ILA types, *p* = 0.04).

**Table 4:** Comparison of CT features

CT features	Post-COVID-19 residual abnormalities (*n* = 20)	Fibrotic ILA (*n* = 20)	Non-fibrotic ILA (*n* = 20)	*p-*value^a^
**Total lesion extent**				
> 25%	14 (70)^b,c^	1 (5.0)	1 (5.0)	0.02
≤ 25%	6 (30)	19 (95)	19 (95)	
**Distribution**				
Subpleural	9 (45)^b,c^	20 (100)	19 (95)	< 0.001
Peribronchovascular	15 (75)^b,c^	1 (5.0)	5 (25)	< 0.001
Basal predominance	7 (35)^b,c^	18 (90)	17 (85)	< 0.001
Posterior predominance	10 (50)^b,c^	19 (95)	17 (85)	0.002
**Dominant findings**				
GGO dominant	12 (60)^b^	3 (15)	16 (80)	< 0.001
Reticulation dominant	8 (40)	17 (85)	4 (20)	
**Other findings**				
Non-emphysema cysts	1 (5.0)^b^	7 (35)	1 (5.0)	0.009
Bronchial dilation	8 (40)^b,c^	8 (40)	3 (15)	< 0.001
Perilobular opacities	15 (75)^b,c^	1 (5.0)	4 (20)	< 0.001
Parenchymal bands	17 (85)^b,c^	3 (15)	4 (20)	< 0.001
Mosaic attenuation	3 (15)	0 (0.0)	0 (0.0)	0.04

Data are numbers of patients, with percentages in parentheses

^a^ The *p*-value is for the comparison among three groups

^b^ Indicates a significant difference compared to fibrotic ILA in a subgroup comparison (*p* < 0.05)

^c^ Indicates a significant difference compared to non-fibrotic ILA in a subgroup comparison (*p* < 0.05)

## Discussion

This study assessed radiologists’ ability to distinguish post-COVID-19 residual abnormalities from ILA. Radiologists showed good diagnostic performance in distinguishing post-COVID-19 residual abnormalities from ILA (median AUC 0.85) with moderate agreement (κ = 0.56). However, radiologists were better at distinguishing post-COVID-19 abnormalities from fibrotic ILA than at distinguishing post-COVID-19 abnormalities from non-fibrotic ILA (median AUC = 0.89 vs. 0.80, *p* = 0.003).

Residual lung parenchymal abnormalities, a form of chronic lung injury following COVID-19, were observed on CT scans in 24% to 54% of patients hospitalized for COVID-19 pneumonia at 1 to 2-year follow-ups [10]. Although the manifestation of parenchymal abnormalities on CT scans varies according to the severity of the initial infection and the time elapsed thereafter [11], commonly observed residual lung abnormalities 1 year after COVID-19 infection include faint GGO, perilobular opacities, reticulation, bronchiectasis, parenchymal bands, crazy-paving appearance, and mosaic attenuation, with some of these findings improving over time [10, 12]. Recent 2- to 3-year follow-up studies [13, 14] have observed a similar trend of slow but gradual resolution of CT abnormalities: GGOs show relative resolution, while irregular lines, reticular opacities, and traction bronchiectasis tend to persist, suggesting that some of these residual findings may correspond to post-infectious fibrosis [13]. On the other hand, CT findings of ILA include ground-glass or reticular abnormalities, lung distortion, traction bronchiectasis or bronchiolectasis, honeycombing, and non-emphysematous cysts with a subpleural and posterior predominant distribution [9]. Our study noted that wider extent, peribronchovascular distribution, perilobular opacities, parenchymal bands, and mosaic attenuation were more common in cases of post-COVID-19 residual abnormalities than in both fibrotic and non-fibrotic ILA. However, subpleural distribution and basal/posterior predominance, as well as non-emphysematous cysts, were less common in cases of post-COVID-19 residual abnormalities. The GGO-dominant finding was more common in post-COVID-19 residual abnormality than in fibrotic ILA but not statistically different compared to non-fibrotic ILA. Based on these results, the extent and distribution of lesions, along with some associated findings, may be helpful in distinguishing features between post-COVID-19 residual abnormalities and ILA.

Most post-COVID-19 residual abnormalities are mild, and a progressive course is not typical [15]. Recent studies suggest that the clinical course and CT findings of COVID-19 pneumonia often resemble those of post-infectious organizing pneumonia. In most cases, improvement in radiological findings and lung function is observed over time, and only a minority have irreversible fibrosis [2, 3, 16, 17]. Similarly, long-term studies of SARS-CoV, Middle East respiratory syndrome, and acute respiratory distress syndrome from other viral causes can also improve over weeks or months [18, 19]. The bronchial dilation observed in post-COVID-19 residual abnormalities often resolves during convalescence, necessitating caution in labeling these cases as traction bronchiectasis until long-term follow-up confirms their irreversibility [20]. Honeycombing, a definitive indicator of fibrosis, is rarely observed in post-COVID-19 residual abnormalities, with most reports indicating a prevalence of < 5% [21, 22]. Uncertainties about the progression to progressive ILD due to COVID-19 remain, but post-COVID-19 residual abnormalities can be monitored through serial follow-up imaging. Therefore, it is important for radiologists to distinguish these findings even in the absence of clinical information in the era of recovery from the pandemic.

In our study, radiologists demonstrated good diagnostic performance in distinguishing post-COVID-19 residual abnormalities from ILA (median AUC, 0.85). However, their performance was lower for distinguishing non-fibrotic ILA than for distinguishing fibrotic ILA from post-COVID-19 abnormalities (median AUC, 0.80 vs. 0.89, *p* = 0.003). Furthermore, the most common misdiagnosis for post-COVID-19 residual abnormalities was non-fibrotic ILA, and conversely, non-fibrotic ILA was most frequently misdiagnosed as post-COVID-19 residual abnormalities. This highlights the difficulties in distinguishing these two conditions and emphasizes the significance of acquiring COVID-19 history, especially if hospitalization from the infection, when non-fibrotic ILA is suspected. Furthermore, if a patient has a documented history of severe COVID-19 or pre-existing residual CT abnormalities following COVID-19, non-fibrotic ILA should be avoided when describing the CT findings, as ILA is, by definition, an incidental finding without previous clinical suspicion of ILD [9].

Our study has several limitations. First, due to the retrospective nature of case inclusion, it was not clearly confirmed whether there had been any abnormalities in the chest CT before COVID-19 for patients with post-COVID-19 residual abnormalities. Additionally, the study did not include a sufficiently diverse range of post-infection periods to explore how changes in post-COVID-19 abnormalities over time might affect diagnostic performance. Second, the evaluation of diagnostic capabilities was exclusively based on representative images, without incorporating clinical context or considering prevalence, which may not accurately reflect real-world scenarios. Third, due to the practical challenges of involving a diverse multinational group of readers with varying levels of experience, the number of test cases was inherently limited. This constraint might have restricted the scope of our findings across a broader spectrum of cases.

In conclusion, radiologists demonstrated good diagnostic performance and moderate interobserver agreement in distinguishing post-COVID-19 residual abnormalities from ILA. Caution is required to avoid misdiagnosing post-COVID-19 residual abnormalities as non-fibrotic ILA, and it may be helpful to consider additional clinical information in cases of uncertainty.

## Supplementary information

ELECTRONIC SUPPLEMENTARY MATERIAL

## Abbreviations

AUC: Area under the receiver operating characteristic curve
ILA: Interstitial lung abnormalities
ILD: Interstitial lung diseases
