# Supplementary material for: Diagnostic performance of radiologists in distinguishing post-COVID-19 residual abnormalities from interstitial lung abnormalities
Source: Eur Radiol. 2024 Sep 23;35(4):2265–74. doi: 10.1007/s00330-024-11075-x (PMC11913901; doi:10.1007/s00330-024-11075-x)
Supplement: Supplementary file 1 — ELECTRONIC SUPPLEMENTARY MATERIAL [file 330_2024_11075_MOESM1_ESM.pdf]

Diagnostic Performance of Radiologists in Distinguishing Post-COVID-19 Residual Abnormalities from Interstitial Lung Abnormalities

ELECTRONIC SUPPLEMENTARY MATERIAL

Supplementary Figure S1. Diagnostic scoring scales.

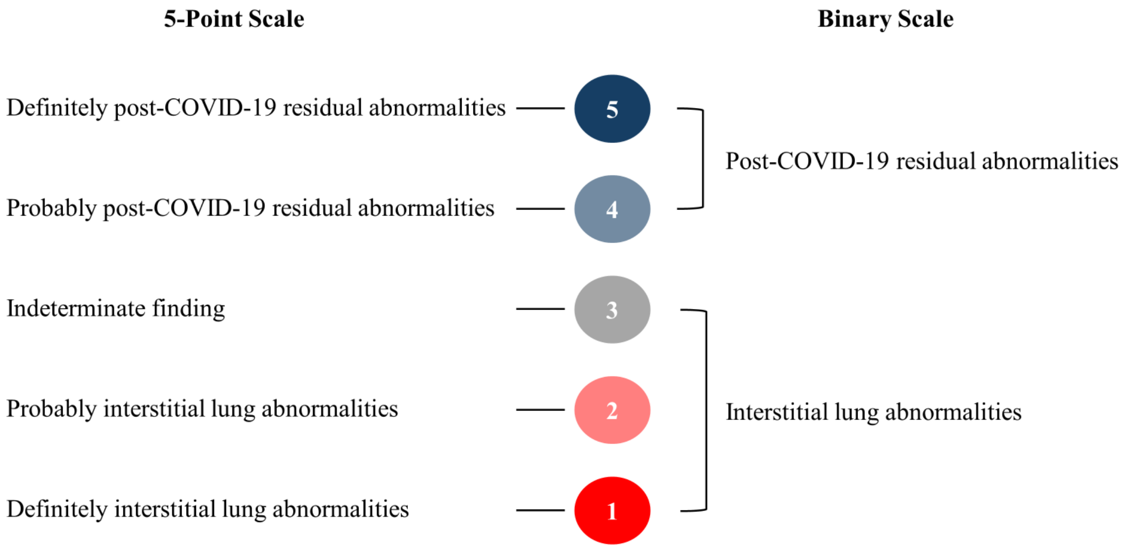

Supplementary Table S1. Interobserver agreement of post-COVID-19 residual abnormalities vs. ILA

Readers	Interobserver agreement			
	5-point scale	*p*-value	Binary scale	*p*-value
**More experienced group**†				
Consensus vs. R1	0.64 (0.48, 0.79)	< 0.001	0.69 (0.50, 0.88)	< 0.001
Consensus vs. R2	0.54 (0.39, 0.70)	<0.001	0.67 (0.48, 0.85)	< 0.001
Consensus vs. R3	0.63 (0.52, 0.75)	< 0.001	0.83 (0.69, 0.97)	< 0.001
Consensus vs. R4	0.69 (0.54, 0.84)	< 0.001	0.49 (0.27, 0.72)	< 0.001
Consensus vs. R5	0.40 (0.24, 0.56)	< 0.001	0.41 (0.19, 0.63)	0.001
Consensus vs. R6	0.74 (0.63, 0.85)	< 0.001	0.69 (0.54, 0.84)	< 0.001
Consensus vs. R7	0.49 (0.36, 0.61)	< 0.001	0.64 (0.44, 0.84)	< 0.001
**Less experienced group**‡				
Consensus vs. R8	0.72 (0.59, 0.84)	< 0.001	0.71 (0.53, 0.90)	< 0.001
Consensus vs. R9	0.57 (0.40, 0.73)	< 0.001	0.61 (0.40, 0.82)	< 0.001
Consensus vs. R10	0.47 (0.34, 0.59)	< 0.001	0.76 (0.59, 0.93)	< 0.001
Consensus vs. R11	0.55 (0.40, 0.69)	< 0.001	0.56 (0.36, 0.77)	< 0.001
Consensus vs. R12	0.63 (0.50, 0.76)	< 0.001	0.86 (0.73, 0.99)	< 0.001
Consensus vs. R13	0.44 (0.27, 0.62)	< 0.001	0.47 (0.25, 0.69)	< 0.001
Consensus vs. R14	0.49 (0.32, 0.65)	< 0.001	0.66 (0.47, 0.85)	< 0.001
Consensus vs. R15	0.47 (0.32, 0.61)	<0.001	0.57 (0.36, 0.79)	< 0.001

Note. - The answer chosen by most readers can be referred to as the "Consensus".

† More experienced readers had 21-33 years of chest CT interpretation experience.

‡ Less experienced readers had 8-20 years of chest CT interpretation experience.

Supplementary Table S2. Diagnostic performance of post-COVID-19 residual abnormalities vs. ILA

Readers	AUC	Accuracy	Sensitivity	Specificity
	5-point scale	Binary scale	Binary scale	Binary scale
**More experienced group**[†]				
R1	0.87 (0.78, 0.97)	0.82 (0.70, 0.90)	0.85 (0.62, 0.97)	0.80 (0.64, 0.91)
R2	0.86 (0.76, 0.95)	0.80 (0.68, 0.89)	0.95 (0.75, 1.00)	0.73 (0.56, 0.85)
R3	0.92 (0.83, 1.00)	0.82 (0.70, 0.90)	0.90 (0.68, 0.99)	0.78 (0.62, 0.89)
R4	0.81 (0.69, 0.93)	0.77 (0.64, 0.87)	0.60 (0.36, 0.81)	0.85 (0.70, 0.94)
R5	0.67 (0.53, 0.81)	0.60 (0.47, 0.72)	0.70 (0.46, 0.88)	0.55 (0.38, 0.71)
R6	0.85 (0.74, 0.95)	0.80 (0.68, 0.89)	0.65 (0.41, 0.85)	0.88 (0.73, 0.96)
R7	0.86 (0.76, 0.97)	0.83 (0.71, 0.92)	0.75 (0.51, 0.91)	0.88 (0.73, 0.96)
**Less experienced group**[‡]				
R8	0.86 (0.76, 0.96)	0.83 (0.71, 0.92)	0.75 (0.51, 0.91)	0.88 (0.73, 0.96)
R9	0.84 (0.73, 0.96)	0.82 (0.70, 0.90)	0.75 (0.51, 0.91)	0.85 (0.70, 0.94)
R10	0.82 (0.70, 0.93)	0.78 (0.66, 0.88)	0.80 (0.56, 0.94)	0.78 (0.62, 0.89)
R11	0.82 (0.71, 0.93)	0.72 (0.59, 0.83)	0.80 (0.56, 0.94)	0.68 (0.51, 0.81)
R12	0.86 (0.76, 0.95)	0.80 (0.68, 0.89)	0.85 (0.62, 0.97)	0.78 (0.62, 0.89)
R13	0.71 (0.58, 0.85)	0.63 (0.50, 0.75)	0.70 (0.46, 0.88)	0.60 (0.43, 0.75)
R14	0.82 (0.71, 0.93)	0.77 (0.64, 0.87)	0.80 (0.56, 0.94)	0.75 (0.59, 0.87)
R15	0.86 (0.76, 0.97)	0.80 (0.68, 0.89)	0.70 (0.46, 0.88)	0.85 (0.70, 0.94)

Note. - Data in parentheses are 95% confidence interval.

[†] More experienced readers had 21-33 years of chest CT interpretation experience.

[‡] Less experienced readers had 8-20 years of chest CT interpretation experience.

Supplementary Table S3. Diagnostic performance of post-COVID-19 residual abnormalities vs. Fibrotic ILA

Readers	AUC	Accuracy	Sensitivity	Specificity
	5-point scale	Binary scale	Binary scale	Binary scale
**More experienced group**†				
R1	0.90 (0.80, 0.99)	0.83 (0.67, 0.93)	0.85 (0.62, 0.97)	0.80 (0.56, 0.94)
R2	0.89 (0.79, 1.00)	0.85 (0.70, 0.94)	0.95 (0.75, 1.00)	0.75 (0.51, 0.91)
R3	0.94 (0.86, 1.00)	0.88 (0.73, 0.96)	0.90 (0.68, 0.99)	0.85 (0.62, 0.97)
R4	0.88 (0.78, 0.98)	0.78 (0.62, 0.89)	0.60 (0.36, 0.81)	0.95 (0.75, 1.00)
R5	0.78 (0.64, 0.92)	0.68 (0.51, 0.81)	0.70 (0.46, 0.88)	0.65 (0.41, 0.85)
R6	0.92 (0.83, 1.00)	0.83 (0.67, 0.93)	0.65 (0.41, 0.85)	1.00 (0.83, 1.00)
R7	0.92 (0.82, 1.00)	0.85 (0.70, 0.94)	0.75 (0.51, 0.91)	0.95 (0.75, 1.00)
**Less experienced group**‡				
R8	0.85 (0.72, 0.97)	0.83 (0.67, 0.93)	0.75 (0.51, 0.91)	0.90 (0.68, 0.99)
R9	0.85 (0.73, 0.97)	0.78 (0.62, 0.89)	0.75 (0.51, 0.91)	0.80 (0.56, 0.94)
R10	0.86 (0.74, 0.98)	0.83 (0.67, 0.93)	0.80 (0.56, 0.94)	0.85 (0.62, 0.97)
R11	0.90 (0.81, 1.00)	0.85 (0.70, 0.94)	0.80 (0.56, 0.94)	0.90 (0.68, 0.99)
R12	0.92 (0.83, 1.00)	0.88 (0.73, 0.96)	0.85 (0.62, 0.97)	0.90 (0.68, 0.99)
R13	0.68 (0.52, 0.85)	0.6 (0.43, 0.75)	0.70 (0.46, 0.88)	0.50 (0.27, 0.73)
R14	0.89 (0.78, 0.99)	0.85 (0.70, 0.94)	0.80 (0.56, 0.94)	0.90 (0.68, 0.99)
R15	0.89 (0.77, 1.00)	0.80 (0.64, 0.91)	0.70 (0.46, 0.88)	0.90 (0.68, 0.99)

Note. - Data in parentheses are 95% confidence interval.

† More experienced readers had 21-33 years of chest CT interpretation experience.

‡ Less experienced readers had 8-20 years of chest CT interpretation experience.

Supplementary Table S4. Diagnostic performance of post-COVID-19 residual abnormalities vs. Non-fibrotic ILA

Readers	AUC	Accuracy	Sensitivity	Specificity
	5-point scale	Binary scale	Binary scale	Binary scale
**More experienced group**†				
R1	0.85 (0.73, 0.97)	0.83 (0.67, 0.93)	0.85 (0.62, 0.97)	0.80 (0.56, 0.94)
R2	0.83 (0.69, 0.96)	0.83 (0.67, 0.93)	0.95 (0.75, 1.00)	0.70 (0.46, 0.88)
R3	0.89 (0.79, 1.00)	0.80 (0.64, 0.91)	0.90 (0.68, 0.99)	0.70 (0.46, 0.88)
R4	0.74 (0.58, 0.90)	0.68 (0.51, 0.81)	0.60 (0.36, 0.81)	0.75 (0.51, 0.91)
R5	0.56 (0.38, 0.74)	0.58 (0.41, 0.73)	0.70 (0.46, 0.88)	0.45 (0.23, 0.68)
R6	0.78 (0.63, 0.92)	0.7 (0.53, 0.83)	0.65 (0.41, 0.85)	0.75 (0.51, 0.91)
R7	0.81 (0.67, 0.95)	0.78 (0.62, 0.89)	0.75 (0.51, 0.91)	0.80 (0.56, 0.94)
**Less experienced group**‡				
R8	0.87 (0.76, 0.98)	0.80 (0.64, 0.91)	0.75 (0.51, 0.91)	0.85 (0.62, 0.97)
R9	0.84 (0.70, 0.97)	0.83 (0.67, 0.93)	0.75 (0.51, 0.91)	0.90 (0.68, 0.99)
R10	0.77 (0.62, 0.92)	0.75 (0.59, 0.87)	0.80 (0.56, 0.94)	0.70 (0.46, 0.88)
R11	0.75 (0.59, 0.90)	0.63 (0.46, 0.77)	0.80 (0.56, 0.94)	0.45 (0.23, 0.68)
R12	0.80 (0.65, 0.94)	0.75 (0.59, 0.87)	0.85 (0.62, 0.97)	0.65 (0.41, 0.85)
R13	0.74 (0.59, 0.89)	0.70 (0.53, 0.83)	0.70 (0.46, 0.88)	0.70 (0.46, 0.88)
R14	0.76 (0.61, 0.90)	0.70 (0.53, 0.83)	0.80 (0.56, 0.94)	0.60 (0.36, 0.81)
R15	0.84 (0.71, 0.97)	0.75 (0.59, 0.87)	0.70 (0.46, 0.88)	0.80 (0.56, 0.94)

Note. - Data in parentheses are 95% confidence interval.

† More experienced readers had 21-33 years of chest CT interpretation experience.

‡ Less experienced readers had 8-20 years of chest CT interpretation experience.
